# A Symbolic AI Approach to Medical Training

**DOI:** 10.1007/s10916-024-02139-y

**Published:** 2025-01-09

**Authors:** Alessio Bottrighi, Federica Grosso, Marco Ghiglione, Antonio Maconi, Stefano Nera, Luca Piovesan, Erica Raina, Annalisa Roveta, Paolo Terenziani

**Affiliations:** 1 Computer Science Institute, DISIT, University of Eastern Piedmont, Alessandria, Italy; 2 Integrated Laboratory of AI and Medical Informatics, DAIRI – SS. Antonio e Biagio e Cesare Arrigo Hospital, DISIT – University of Eastern Piedmont, Alessandria, Italy; 3 Mesothelioma, Melanoma and Rare Cancers Unit, Azienda Ospedaliera “SS Antonio e Biagio e Cesare Arrigo”, Alessandria, Italy; 4 Skin Cancer Unit, Azienda Ospedaliera “SS Antonio e Biagio e Cesare Arrigo”, Alessandria, Italy; 5 Research Innovation Department, Research Training Innovation Infrastructure Azienda Ospedaliera “SS. Antonio e Biagio e Cesare Arrigo”, Alessandria, Italy

**Keywords:** Educational knowledge-based AI system, Computer-interpretable clinical guidelines, Knowledge representation, Medical training and assessment, Clinical guideline simulation

## Abstract

In traditional medical education, learners are mostly trained to diagnose and treat patients through supervised practice. Artificial Intelligence and simulation techniques can complement such an educational practice. In this paper, we present GLARE-Edu, an innovative system in which AI knowledge-based methodologies and simulation are exploited to train learners “how to act” on patients based on the evidence-based best practices provided by clinical practice guidelines. GLARE-Edu is being developed by a multi-disciplinary team involving physicians and AI experts, within the AI-LEAP (LEArning Personalization of AI and with AI) Italian project. GLARE-Edu is domain-independent: it supports the acquisition of clinical guidelines and case studies in a computer format. Based on acquired guidelines (and case studies), it provides a series of educational facilities: (i) *navigation*, to navigate the structured representation of the guidelines provided by GLARE-Edu, (ii) *automated simulation*, to show learners how a guideline would suggest to act, step-by-step, on a specific case, and (iii) (self)*verification*, asking learners how they would treat a case, and comparing step-by-step the learner’s proposal with the suggestions of the proper guideline. In this paper, we describe GLARE-Edu architecture and general features, and we demonstrate our approach through a concrete application to the melanoma guideline and we propose a preliminary evaluation.

## Introduction

Traditional medical texts and courses cover a wide range of knowledge, ranging from human anatomy to the description of diseases, and their diagnosis and treatment. However, “operational” problems such as “***how to act on a specific patient?***”, “***How to diagnose and treat a patient by applying the best medical procedures?***” are fundamental in medical education, thus deserving specific attention and dedicated methodologies and tools. As a matter of fact, the knowledge about the “best” evidence-based clinical procedures to diagnose and treat patients affected by a specific disease is widely available, textually encoded into clinical practice guidelines (CPGs). CPGs are “*systematically developed statements to assist practitioner and patient decisions about appropriate healthcare under specific clinical circumstances*” [1]. CPGs are widely promoted by national and international healthcare agencies, to introduce evidence-based medicine and support the quality and standardization of healthcare services. Thousands of textual CPGs are currently available (e.g., the Guideline International Network [2] provides more than 6500 CPGs). However, many CPGs are encoded in textual form as books of several hundred pages. Thus, it is very difficult to learn them and to select from them exactly the recommendation suitable to cope with a specific patient. For instance, a recent need-assessment of CPGs in medical education carried on by the Guideline International Network working group about medical education pointed out that the majority of medical students encounter guidelines for the first time in their clinical years, and are often unable to effectively use CPGs typically due to a lack of education. A main critical issue pointed out by such an analysis is the *need of supports for education on the proper use of CPGs* [3]. Computer-based approaches can play a relevant role in this context: “*The literature reveals that Clinical Informatics training in the core curricula will complement if not enhance clinical skills*”, so that “*future research needs to comprehensively address current gaps in Clinical Informatics training in different contexts*” [4]. However, a recent analysis has shown that, though learners showed higher satisfaction with digital education compared to traditional learning, the impact of current educational digital techniques in the context of CPG is still quite limited [5]. Notably, in the area of CPGs, since the 1980s, the AI in Medicine (AIM) community has started to design domain-independent systems to *acquire* CPGs in a computer-interpretable format, and to support their *execution* on specific patients (see, e.g., the surveys [6–8]). GLARE (Guideline Acquisition Representation and Execution) is one of such systems, which we have been developing and adopting since 1996 [9]. However, such systems are mainly devoted to the ***decision support*** task, while only few CPG-based AIM approaches have started to investigate the educational task (see Sect. 5). In this paper, starting from our long-term GLARE experience, we propose a new system, GLARE-Edu, specifically devoted to medical education. GLARE-Edu is *domain-independent*: it is not devoted to a specific CPG, but supports the acquisition and use of different CPGs. Since CPGs are collections of well-defined and quite structured explicit knowledge, GLARE-Edu is characterized by the development/adoption of *symbolic AI techniques* (e.g., *knowledge representation*,* planning*,* logic-based reasoning*,* temporal reasoning*) to represent and operate on it (as opposed to Machine Learning techniques, suitable when large amounts of data are available)^1^.

GLARE-Edu provides a tool to support medical experts in the ***acquisition*** of CPGs in a computer format, henceforth called *Computer-Interpretable Guidelines* (**CIG**). An additional tool supports the acquisition of (virtual) ***case studies***, intended as the computer-interpretable description of the evolution of the patients’ clinical parameters/data. The core of GLARE-Edu consists of the ***navigation***, ***automated simulation***, and ***verification*** tools, using the acquired CPGs and case studies to address different aspects/competencies of medical education.

We are developing GLARE-Edu within a two-year project, AI-LEAP (LEArning Personalization of AI and with AI) [11], in a multi-disciplinary team including members from our University and from the hospital SS. Antonio, Biagio e Cesare Arrigo in Alessandria. In this paper, we describe the results of the first year of the project. In Sect. “GLARE-Edu: background, underlying philosophy, educational impact” we highlight the background, the underlying philosophy, and the educational impact of GLARE-Edu. In Sect. “GLARE-Edu: architecture and main features” we show the overall architecture of GLARE-Edu, and discuss its components. In Sect. “Evaluation of GLARE-Edu’s Educational Approach”, we sketch a preliminary evaluation of our approach and the evaluation plan provided in the AI-LEAP project. Section “Related work” discusses related work, and Sect. “Conclusions and future works” presents conclusions and future work.

## GLARE-Edu: Background, Underlying Philosophy, Educational Impact

In this section, we motivate the need for educational CIG systems like GLARE-Edu (with respect to existent CIG systems for decision support; subsection “CIG-based clinical decision support systems vs. educational systems”), present GLARE-Edu underlying principles (subsection “GLARE-Edu underlying philosophy”), and discuss the main tasks of GLARE-Edu and its educational impact (subsection “Tasks and educational impact”).

### CIG-Based Clinical Decision Support Systems vs. Educational Systems

As already mentioned in the introduction, given the role of CPGs in medical practice, many different domain-independent ***decision support*** systems have been built to acquire CPGs in computer format (i.e., into a CIG) and to support physicians in their application to patients (see, e.g., the surveys [6–8]). Since CPGs consist of large bodies of well-defined and quite structured knowledge, and physicians appreciate an explicit representation and management of such a knowledge, such systems are characterized by the adoption of advanced ***symbolic*** AI methodologies (e.g., in GLARE: *knowledge representation*,* ontological reasoning*,* temporal reasoning*,* decision theory*,* model-based verification*,* conformance evaluation*) to acquire and represent it, and to provide user-physicians with different forms of support [12, 13].

1. **CIG Decision Support Systems Behavior**: In short, once a CIG is acquired, such systems take as input it and the clinical record of a *patient* (managed by an external DBMS), and, step-by-step, automatically determine the next actions or decisions to be taken for the patient, according to the CIG. Step-by-step, actions and decisions are suggested to the physician, who decides whether accepting and applying them to the *patient* or not.

Though some of such systems have also been used for educational purposes, their behavior is geared towards to decisions support. In GLARE-Edu, we support (besides other facilities) two main behaviors (different from the above).

#### CIG Educational System (GLARE-Edu) Behaviors

First of all, for education, a system cannot connect with DBMS managing clinical records, but has to acquire a-priori also *case studies* (besides CIGs). Given a CIG and a case study for it, GLARE-Edu provides (B2) and (B3).

(B2) **GLARE-Edu’s Automated Simulation**. Step-by-step, GLARE-Edu automatically determines the next actions or decisions to be taken to manage the *case study*, according to the CIG. Actions and decisions are shown step-by-step to the learner, to demonstrate how the CIG recommends to manage the case study (notably, differently from (B1), in automated simulation, the system “leads”, and the user cannot make choices).
(B3) **GLARE-Edu’s Verification**. Step-by-step, GLARE-Edu lets the learner propose how he or she would act on the case study, and automatically checks whether his or her proposal is consistent with what the CIG would have recommended to do, providing explanations in the case of differences (notably, such a behavior is completely different from (B1), and GLARE-Edu is the first CIG system in the literature providing it).

### GLARE-Edu Underlying Philosophy

GLARE-Edu grounds on the 25-year work of (part of) the team about GLARE, a CIG system devoted to *decision support* [9]. GLARE-Edu builds on top of GLARE, moving the attention from decision support to education, but maintaining its basic underlying principles, i.e., being.

- ***Domain-independent***: in GLARE, we have acquired and managed many different CPGs, including asthma, ischemic stroke, alcohol-related diseases, gastro-esophageal disease, polytrauma.
- ***User-friendly***: GLARE’s (and GLARE-Edu’s) representation formalism and its facilities have been designed and tested by a multidisciplinary team including physicians.
- A ***support system***: GLARE (and GLARE-Edu’s) is intended to help users, and not to substitute them.

In particular, GLARE-Edu basically adopts the same representation formalism used by GLARE, so that it takes advantage of GLARE’s acquisition (see Sect. 3.3) and navigation (see Sect. 3.5) tools, and has been designed in cooperation with physicians that are involved also in its educational use (consider, e.g., the experimental evaluation described in Sect. 4).

### Tasks and Educational Impact

**Fig. 1:**
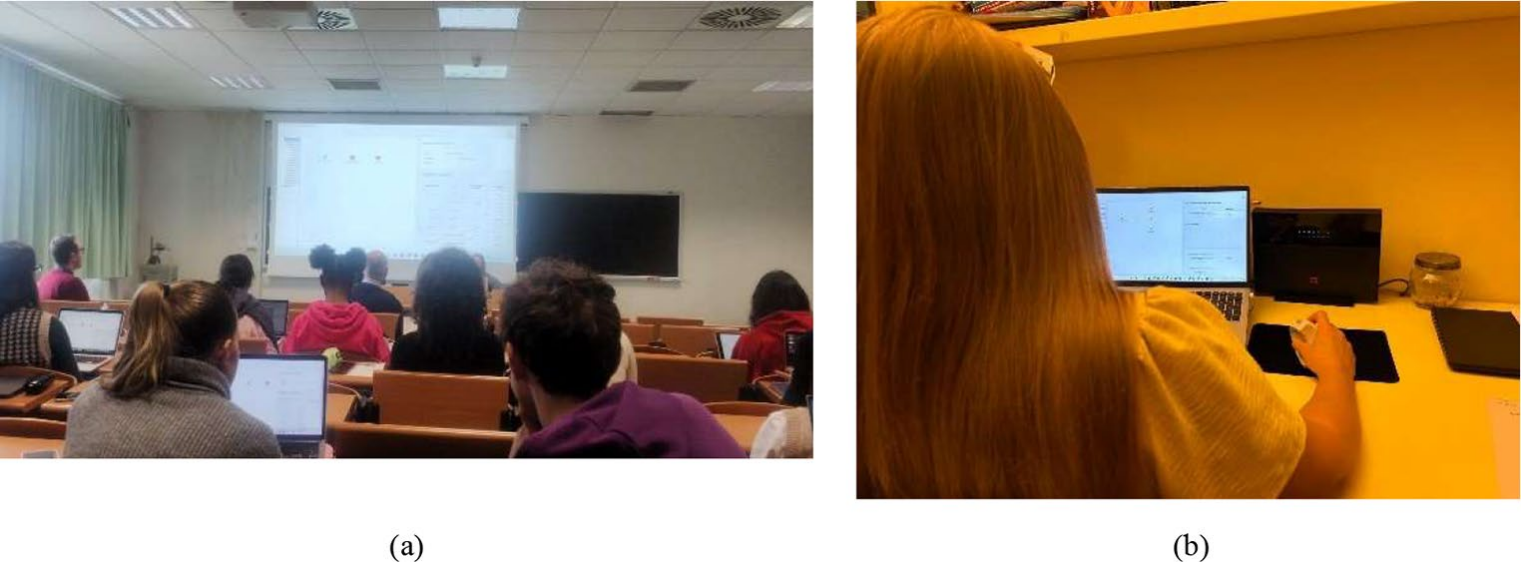
Examples of the two different modalities of use of GLARE-Edu: supervised (**a**) and non-supervised (**b**)

To clarify the goals and educational impact of GLARE-Edu, it is important to clarify that it supports two different modalities of use:

- ***Supervised*** use, in which lessons are held by medical experts, who adopt GLARE-Edu navigation, automated simulation and verification facilities to enrich their lessons (see Fig. 1a).
- ***Non-supervised*** use, in which the above facilities are directly used by learners, to facilitate their learning process (see Fig. 1b).

GLARE-Edu supports three main educational tasks. For each of them, we discuss GLARE-Edu’s educational impact, considering both its supervised and non-supervised use, and comparing it with “traditional” learning (i.e., learning without an AI system support).

#### Task 1: Learning a CPG “per se”

The first step for learners is obviously to learn the content of a general CPG, independently of its application to specific case studies. Notably, a CPG is usually a quite “flat” text of hundreds of pages, and, in “traditional” learning, the teacher is left alone to organize its presentation to learners, while learners have to study such “flat” text with the only support of teacher’s explanations (and possibly, teacher-provided additional material). GLARE-Edu provides a **navigation** facility to support them. Notably, a CIG (i.e., the computer version of a CPG, as acquired by GLARE-Edu) can be roughly assimilated to a “*conceptual map*” of the knowledge expressed in the textual CPG (see, e.g., Fig. 3 in the following), organized at different levels of detail (see the description of GLARE-Edu formalism in Sect. 3.2). GLARE-Edu’s ***navigation*** facility supports the “exploration” of such a conceptual map at different levels of detail (top-down refinement methodology), and can be exploited by teachers (in the supervised modality) as a support to show and discuss the content of a CPG is a structured and well-organized way, and by students (in the non-supervised modality) to access it in the same way.

#### Task 2: Learning How a CPG Recommends to Manage a Case Study

In traditional education, given a (hundred-pages) CPG, a teacher is left alone to show learners which parts of it are relevant to the management of each specific case study, and, in their autonomous study, learners have no support to understand which CPG recommendations applies to specific case studies. GLARE-Edu’s ***automated simulation*** facility performs inferences on the knowledge representation in order to automatically “adapt” the “conceptual map” (i.e., the CIG’s representation) to each specific case study, showing the parts relevant for it. This is a fundamental support for teachers (in the supervised modality) to show and discuss how a general CPG applies to a specific case study, and by learners (in the non-supervised modality) learn that in a facilitated way.

#### Task 3: Verifying Learner’s Ability to Manage a Case Study (with Explanations)

In traditional education, learners’ self-verification is not supported, and the teacher has no support to define case studies, ask learners how they would manage them (given a CPG), and evaluating (with respect to CPG’s recommendations) and explaining learners’ solutions. GLARE-Edu ***verification*** facility is an innovative (the first one in the literature, considering the CPG context) to support teachers (supervised modality) in such a task, and, above all, to support learners’ self-verification. We believe that this is a crucial advance with respect to the state-of-the-art of CIG systems.

Finally, please notice that, to exemplify GLARE-Edu’s behavior and educational impact, we include, as additional material, a short demo showing GLARE-Edu “in action” [14]^2^.

## GLARE-Edu: Architecture and Main Features

In this Section we present the main technical features of GLARE-Edu. We first show the general architecture and representation formalism, and then we present each one of the components of GLARE-Edu’s architecture.

### GLARE-Edu Architecture

**Fig. 2:**
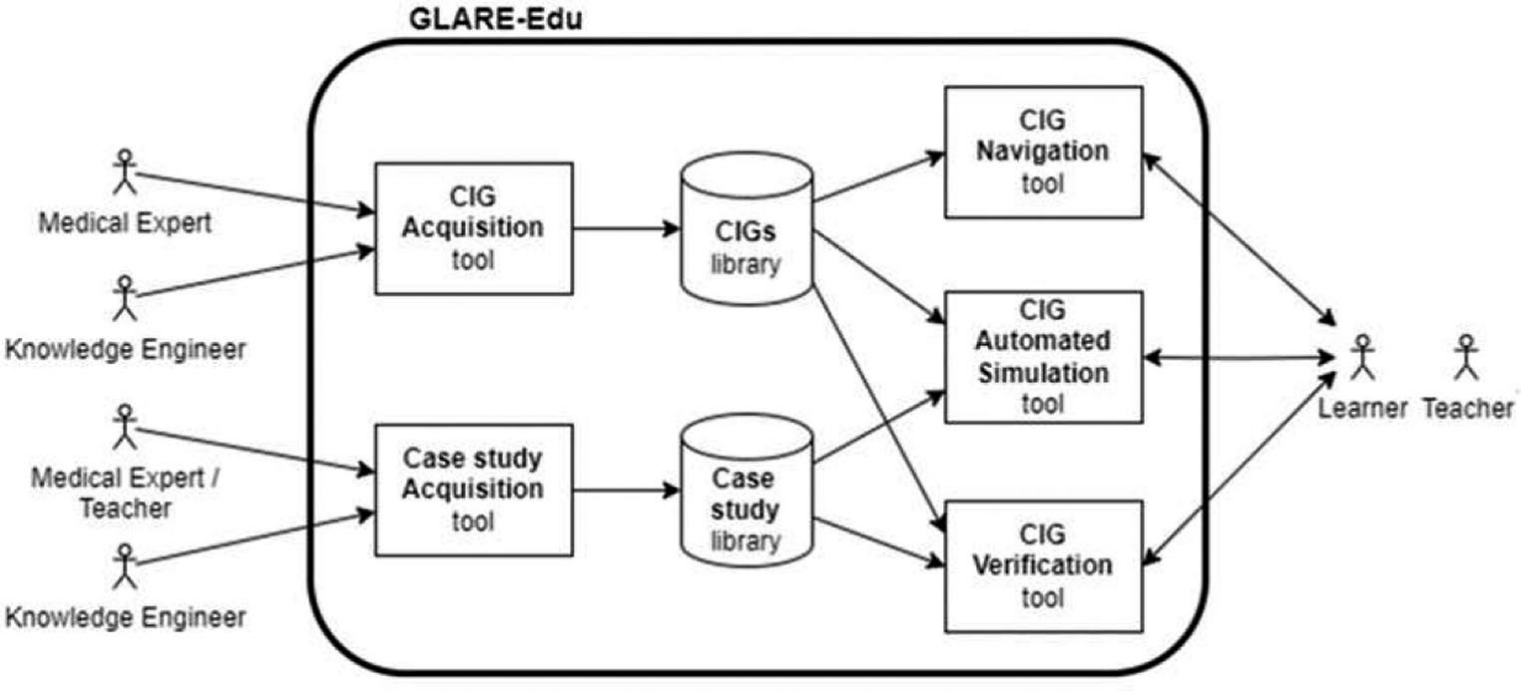
Architecture of GLARE-Edu

Figure 2 illustrates GLARE-Edu’s general architecture. We describe each component in Sect. 3.3–3.7, first with a high-level overview, then discussing key technical challenges, including adapting GLARE for educational use in GLARE-Edu. This contribution may benefit others working on CIG *decision support systems* who are interested in *educational applications* (consider, e.g., [6–8]). For clarity and conciseness, Fig. 2 omits each component’s refined user interface, which we designed and tested in collaboration with physicians from our team, some of whom also serve as teachers in our experimental evaluations.

### CIG Representation Formalism

**Fig. 3:**
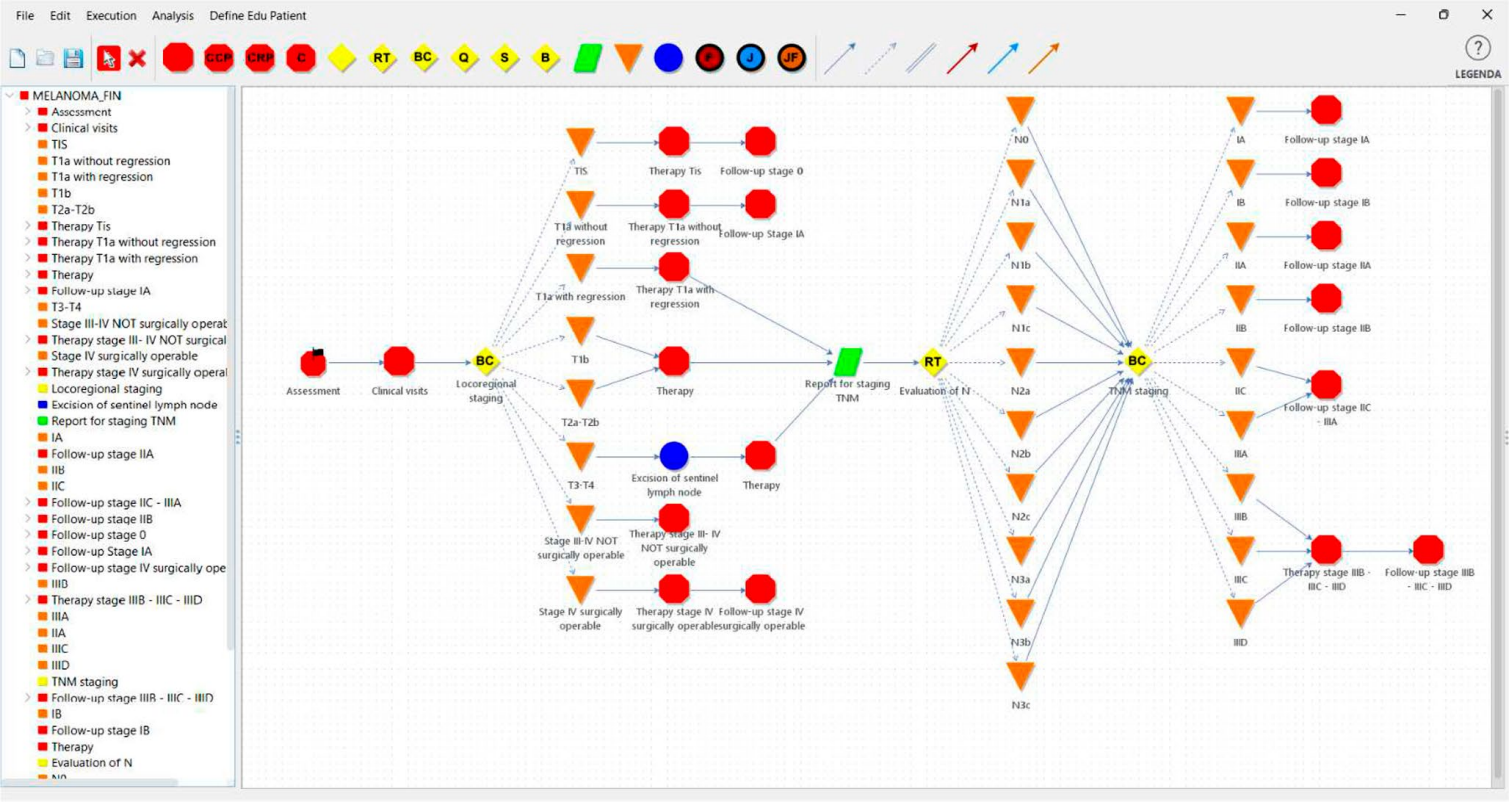
The top level of melanoma CIG (GLARE-Edu graphical interface)

In GLARE-Edu (as in GLARE), a CPG is represented as a hierarchical graph with nodes as actions and arcs as control flow, dictating action order. Nodes and arcs come with descriptive properties. GLARE differentiates between ***atomic*** and ***composite*** actions. ***Composite*** actions, shown as red octagonal nodes (e.g., “Assessment” in Fig. 3), are sub-graphs, aiding hierarchical CPG formalization. Atomic actions represent basic CPG steps, categorized into four types: ***Work actions*** (blue circles, e.g., “Excision of sentinel lymph node”), ***Data requests*** (green parallelograms, e.g., “Report for staging TNM”), ***Decision actions*** (yellow diamonds, e.g., “Locoregional staging”), and ***Conclusions*** (modeling the output of decisions; orange triangles, e.g., “TIS”). Figure 3 shows the Italian CPG for melanoma (released by the Associazione Italiana Oncologia Medica) [15], structured in GLARE-Edu during the project’s first year, offering learners a structured CPG knowledge approach beyond flat textual recommendations.

### CIG Acquisition Tool

GLARE-Edu utilizes GLARE’s acquisition tool, initially detailed in [9].

#### Technical Aspects

In addition to a user-friendly interface for creating the hierarchical CPG graph (see Fig. 3) and entering node descriptions, the tool interacts with a medical ontology (in our implementation, SNOMED CT [16]) to standardize terminology. It also uses advanced symbolic AI techniques, like temporal reasoning, to ensure the consistency of the acquired graph [17].

### Case Study Acquisition Tool

To apply CIGs to practical cases, we need case studies in digital format. Our goal is to teach learners which actions to take for specific cases, not how to perform them. Therefore, virtual reality representation is unnecessary and patients are represented by data tracking clinical status changes.

#### Technical Aspects

Case study creation is a key distinction of GLARE-Edu from GLARE. In GLARE, patient data are managed by an external DBMS, with entries added as new findings appear. Conversely, GLARE-Edu requires “a priori” complete case studies to support simulations (Sect. 3.6 and 3.7). Our case study acquisition tool supports expert physicians in the acquisition: a graphical interface shows them the graph modeling the CIG, and supports step-by-step navigation along *one* (in the case of automated simulation) or *more* (for verification) paths of the given CIG, asking the experts the data values for the case under examination at each data request node.

### CIG Navigation Tool

**Fig. 4:**
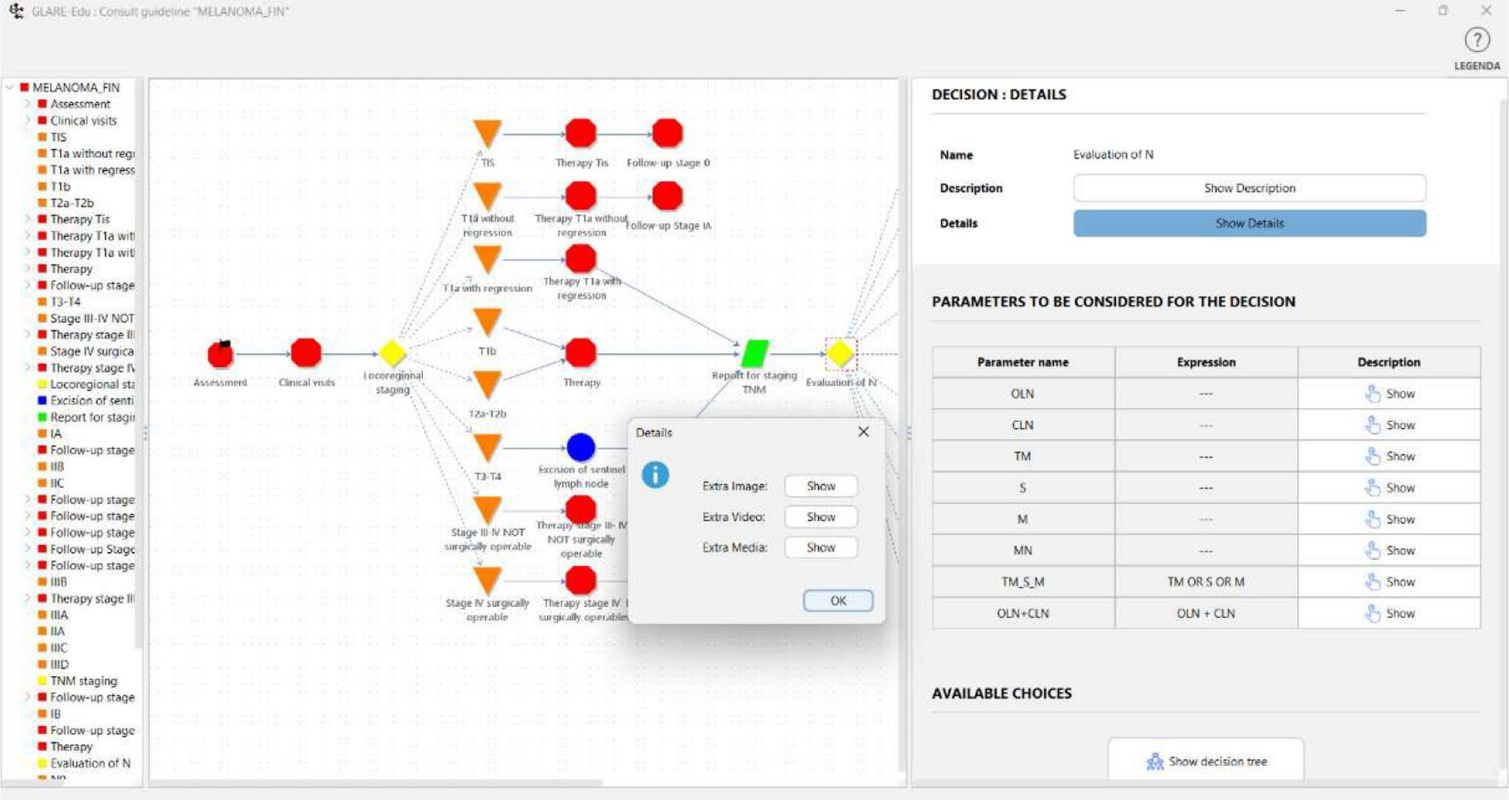
Navigation of the melanoma CIG (GLARE-Edu graphical interface)

GLARE-Edu provides an interactive tool to navigate the hierarchical graph and show the description of nodes. For example, Fig. 4 illustrates the graphical interface for the navigation of the “Evaluation of N” node. Since it is a decision node with a restricted decision tree, learners can easily understand the parameters involved in making the decision and view the structure of the decision tree itself by clicking the “Show decision tree” button. The graphical interface also provides, for each node, a description and additional relevant information that enhances the educational experience. Specifically, by selecting “Show Details”, learners can view the pop-up displayed in Fig. 4, where they have access to supplementary media, such as images, videos or files, through the corresponding button.

#### Technical Aspects

GLARE-Edu CIG navigation is a slight variation (due to minor differences in the graphical interface) of GLARE’s consultation module [9]. Notably, given the hierarchical representation of CIGs, the CIG navigation tool supports the top-down refinement methodology in learning CPG’s contents.

### CIG Automated Simulation Tool

**Fig. 5:**
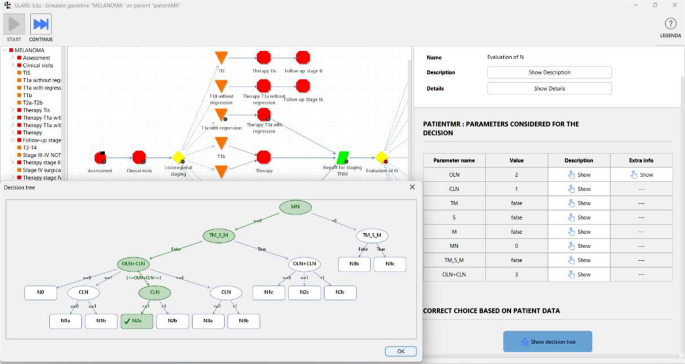
Automated simulation of the melanoma CIG on a case study (GLARE-Edu graphical interface). Notably, the rightmost part of the window shows the case study values

The automated simulation tool uses a CIG and a case study to show, step-by-step, how the CIG would guide actions for the case. Users don’t make choices during this process. For data requests, the tool displays required clinical data, and for decisions, it highlights the correct choice based on criteria and case data (pulled automatically from the stored case). Figure 5 shows a simulation: grey bullets mark completed actions (e.g., from “Assessment” to “Report for staging TNM”), and a red bullet marks the current action (e.g., “Evaluation of N”), with a pop-up showing action details like, in the example, the decision tree modelling the decision and the correct evaluation in it.

#### Technical Aspects

Automated simulation uses GLARE’s execution module, advancing step-by-step along the CIG path based on patient data. Unlike GLARE, where user-physicians can override recommendations, GLARE-Edu only shows CIG recommendations, with no option to override. Additionally, GLARE-Edu operates on a pre-acquired case study, rather than interacting with a DBMS of clinical records.

### CIG Verification Tool

Given a case study, GLARE-Edu lets learners practice applying a CIG, verifying each step against the CIG’s recommendations and offering explanations for discrepancies. For example:

- For **data requests**, GLARE-Edu asks the learner to specify the required data and checks it against the CIG’s data requirements;
- For **clinical decisions**, GLARE-Edu presents the case status and possible outcomes, asking the learner to choose. This answer is compared to the CIG’s decision outcome; if incorrect, an explanation is provided.

**Fig. 6:**
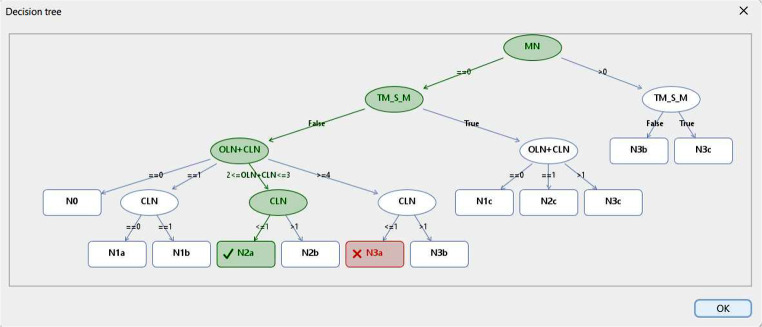
The explanation provided for the decision tree in action “Evaluation of N”, considering the data in the case study (shown in Fig. 5) and the learner’s answer

Figure 6 illustrates an “Evaluation of N” decision example, where the learner’s choice of N3a differs from the correct answer, N2a, and the decision tree shows why. The green nodes represent the tree nodes visited considering the data of the case study, which lead to the correct answer. The leaf corresponding to the learner’s answer is colored in red.

#### Technical Aspects

This verification facility is unique to GLARE-Edu. It balances giving learners freedom to make decisions with the complexity of pre-defining case studies and automatically explaining differences from CIG recommendations. Learners progress step-by-step, choosing actions from a menu; GLARE’s execution module then compares each choice with the CIG’s recommendation, explaining any inconsistencies (e.g., as shown in Fig. 5).

## Evaluation of GLARE-Edu’s Educational Approach

In this section, we outline the evaluation of our approach in two steps:

(i) First (Sect. 4.1) we propose a preliminary evaluation we have already run. We have organized a lesson for physicians focused on the melanoma CPG and selected case studies in collaboration with Alessandria Hospital. The results provide valuable insights into the potential of our approach;
(ii) Second (Sect. 4.2) we detail the plan for our final evaluation: at the end of the AI-LEAP project, we will run two six-month courses to further evaluate — one designed for medical students and another for physicians.

### Preliminary Evaluation

The Alessandria Hospital recruited 20 physicians, for a two-hour lesson considering the melanoma CPG and selected case studies. None of the physicians in the experiment had prior specific knowledge about the Italian melanoma guideline [15]. We present the results concerning the learning phase and the evaluation of GLARE-Edu, since they provide a hint of the potentialities of GLARE-Edu.

The experiment was conducted by splitting the physicians into two homogeneous classes, one adopting GLARE-Edu, and the other receiving traditional medical education. Both classes had an hour and a half lesson, concerning:

1. Specific Parts of the guideline, including the N staging decision.
2. The application of (parts of) the guideline, and of N staging, to specific cases.

For the *control class*, the lesson has been held traditionally, while in the class using GLARE-Edu, the facilities described in Sect. 3 have been exploited. Finally, both classes spent the last 30 min to completing evaluation questionnaires implemented through Google Forms.

We asked the physicians a series of questions, regarding different case studies, asking (i) which (guideline) activities should be performed, (ii) which data should be considered, and (iii) N-staging decisions. For each question, different answers were proposed, and the physicians were asked to choose one of them. Results are summarized in Table 1.

**Table 1:** Percentage of correct answers

	Activities	Required data	*N* staging
Traditional	41%	77%	12.5%
GLARE-Edu	51%	81%	22%

Together with domain-expert teachers, we have analyzed these results and tried to interpret them. The low percentage of correct answers is probably due to several factors: (i) the complexity of the guideline, (ii) the fact that physicians had no specific a-priori knowledge of it, since the management of melanoma patients is not carried on by General Practitioners, but by specialists, and (iii) the short time of the lesson. Notably, the adoption of GLARE-Edu has improved the training results, especially regarding the actions to be performed on patients, and the decision about N staging. The *p-value* for the comprehensive test is 0.348 (since we are in a context of two dichotomous nominal variables and small samples we evaluated the *p-value* through Fisher’s exact test). We are fully aware that this is not a statistically relevant result, but we strongly believe that the low statistical significance is mostly due to (i) the limited number of physicians (and of questions) and (ii) the very short time we could devote to education.

We are confident to achieve better and significant results in our future experimental evaluation at the end of the AI-LEAP project (see Sect. 4.2).

We asked a set of questions exclusively to the GLARE-Edu classes to evaluate the physicians’ perception of its usefulness. Scores ranged from 1 (“minimum”) to 5 (“maximum”). The average values are shown in Table 2.

**Table 2:** A general evaluation of GLARE-Edu. Scores are numeric values (1,2,3,4,5), where 1 stands for “minimum” and 5 for “maximum”

	Average score
Complexity of training about GLARE-Edu representation and use	3.33
Usefulness of GLARE-Edu	4
Would you recommend the use of GLARE-Edu?	4.11

Notably, physicians found “medium” (3,33 is basically a medium value on a scale of 1–5) the extra effort needed to learn GLARE-Edu, and found it useful and “to be recommended”.

### Evaluation Plan (AI-LEAP Project)

The initial results are promising, but the evaluations conducted are quite limited.

As discussed before, the development of GLARE-Edu is within a two-year project, AI-LEAP [11], in which we have established a detailed plan for GLARE-Edu’s evaluation. In particular, the final six months of the AI-LEAP project will be dedicated to an extensive experimental assessment, in which we aim to demonstrate both (i) the generalizability and (ii) the potential of GLARE-Edu.

Regarding (i), we are collaborating with Prof. Luigi Castello, associate professor of internal medicine at the University of Eastern Piedmont and head of Internal Medicine Department 1 at Alessandria Hospital, to acquire a CPG about dyslipidemia, and many case studies for it.

To demonstrate (ii), we are organizing two courses, each one distributed in a six-month period of time (the last six months of the AI-LEAP project):

(A) A course for physicians on Italian melanoma guideline [15];
(B) A course for student in medicine on dyslipidemia based on European Society of Cardiology guidelines [18].

We aim to enroll at least 50 students and 20 physicians.

The two courses are organized in a similar way. We will split *learners* into two homogeneous classes: one adopting GLARE-Edu and the other (the control group) undergoing traditional medical education based on the textual CPGs. Both classes will attend in-person lessons led by expert physicians (in GLARE-Edu class, teachers will take advantage of GLARE-Edu), and at-home exercises (the GLARE-Edu learners will have the opportunity to adopt the system also at home). The abstract schema of lessons is:

- 2 lessons on the CPG;
- A first *in-itinere* evaluation test;
- 2 lessons on the CPG;
- A second *in-itinere* evaluation test;
- 2 lessons on the CPG;
- The final evaluation test.

The course for physician (A) will be organized in collaboration with Alessandria Hospital, which is in the regional register of accredited providers for education since 2012. It will be accredited as Continuous Medical Education (CME) course, which are mandatory for maintaining professional licenses.

The course for student in medicine (B) will be organized in collaboration with our university’s Master Degree in Medicine and Surgery as an “Attività didattiche elettive” (ADE or elective educational activities). ADE are educational activities designed to deepen specific knowledge and skills, enhancing both preparation and training. They provide to students (of the fourth and fifth years) opportunities to explore topics beyond the core curriculum and promote multidisciplinary approaches.

Notably, both courses need a specific accreditation process before the enrollment phase can start^3^.

Furthermore, the AI-LEAP project mandates that our activities during the evaluation phase are conducted under the constant supervision of the Bruno Kessler Foundation [20], serving as external evaluator.

## Related Work

The area of informatics for medical education is wide and complex, and many different surveys are reported in the literature. In the following, we briefly propose a (necessarily partial) overview of some of the main methodologies and applications, with specific focus on the ones more closely related to our approach.

Virtual patients and simulation are certainly one of the areas in which Informatics has provided major contributions. Virtual patients are “*interactive computer simulations of real-life clinical scenarios for health professions training*,* education*,* or assessment*” [21]. Virtual patients differ in terms of competencies (e.g., clinical reasoning, patient communication skills, procedural and basic clinical skills) and technologies (e.g., multimedia system, conversational character, virtual world). The predominant class is the interactive patient scenario followed by software simulations and virtual standardized patients. Other forms of Virtual patients are games, case presentations, human standardized patients, and high-fidelity manikins [22, 23].

Already in the 1980’s, McDowell et al. evaluated the outcomes of simulation methods in nursing education [24]. Recent analyses have shown that virtual patient simulation improved clinical reasoning abilities of medical students in the context of a traditional teaching/learning environment [25], AI virtual patient scenarios were well accepted by students and demonstrated significant promise as a complementary simulation-based learning modality [26], and virtual patient education improved diagnostic performance through deliberate practice [27].

Many of the above approaches also include AI techniques. In the area of medical AI, a large number of approaches was successfully devoted to medical education. For instance, a recent survey [28] identifies many different perspectives for educational applications of AI: Intelligent Tutoring Systems, AI-assisted learner assessment, ChatBot, Personalized Learning Platforms, Robot-Assisted Surgery Simulations, Enhanced anatomy education, AI tools to prepare applications, AI-generated art (to enhance visual storytelling for patient encounters), machine learning for intra-operative video analysis to improve patient care. Several other application contexts have been identified in [29, 30], where also future perspectives are considered.

Notably, a wide range of applications has emerged quite recently, due to the advances of research in the area of Machine Learning (ML) and Deep Learning (DL) [31, 32]. In such a field, the rising of explanation techniques has made ML and DL techniques more exploitable for education [33].

ML and DL have significantly advanced medical education across multiple domains, such as image processing [34–36]. Such technologies assist students through advanced and interactive 3D models [37], and in recognizing and diagnosing medical images such as X-rays, CT scans, and MRIs, providing accurate feedback [38]. These systems are instrumental in teaching students how to identify subtle diagnostic clues.

ML and DL also play a crucial role in enhancing diagnostic reasoning. Tools like CaseB-Pro [39] use neural networks combined with case-based reasoning to help students input patient symptoms and receive diagnostic feedback, improving clinical skills through interactive, real-world simulations. Virtual patient and digital twin systems offer safe environments for students to practice consultations and therapeutic procedures, make diagnostic decisions, and receive instant feedback [40]. Gamification platforms [41] further engage students by adapting learning challenges to their skill level, enhancing both clinical reasoning and patient interaction.

Moreover, adaptive learning systems and Intelligent Tutoring Systems [42] tailor instruction to individual learning needs, ensuring targeted and efficient knowledge acquisition. AI-based assessments [43] provide objective measures of student performance, helping educators identify areas that need improvement.

In the very last years, *generative* AI approaches have added a new dimension to medical education, by simulating patient encounters, answering complex medical questions, and offering personalized feedback in real time [44]. Such AI models enhance clinical reasoning, communication skills, and provide accessible, flexible learning solutions that are revolutionizing medical education.

Additional descriptions of the use of ML and DL for medical education can be found in the surveys [29, 45].

Many other AI educational systems rely on *symbolic* AI methodologies, which are particularly suited to model logical inferences on compositional and structured representations of explicit knowledge [10]. The mere availability of explicit knowledge (as, for example, in medical ontologies like SNOMED CT and UMLS) has proven effective in improving the students’ critical thinking skills [46].

Moving to more complex symbolic AI techniques, one of the most famous and typical applications of symbolic AI methodologies to medicine is the so called expert systems [47], addressing the problem of modeling the diagnostic and/or therapeutic treatment of specific diseases based on an explicit symbolic representation of experts’ knowledge. Most of them have also been used for educational purposes, and, later, have evolved into task-specific educational tools. For instance, the famous MYCIN system was based on a knowledge base of experiential rules (modeled as production rules) and used backward chaining reasoning to support diagnosis and treatment of bacterial infections [48]. It then evolved into the educational systems NEOMYCIN [49] and GUIDON [50].

GIDEON [51] is mainly an expert system for infectious diseases, but it can also be used as a medical education tool. Medical students can use the system to simulate real-world cases and test their diagnostic skills. It uses a rule-based Bayesian reasoning approach.

DXplain [52] is an expert system designed to suggest a list of diseases that are associated with a set of clinical findings entered by a health profession student or practitioner widely adopted also in education.

INTERNIST-I [53] was designed to provide computer-assisted diagnosis in general internal medicine by attempting to model the reasoning of clinicians. It involves a complex diagnostic algorithm, based on explicit knowledge including a hierarchy of disease categories and a heterogeneous set of properties. The Computer-based Patient Case Simulator project was built on top of INTERNIST-I to provide a tool for teaching diagnosis to medical students [54].

The success of expert-system-based approaches in medical education has been relatively limited, largely due to problems in extracting expert knowledge and identifying key evidence-based practices for students. However, such limitations can be addressed with approaches based on computerized clinical guidelines (CIGs), as they exploit widely available evidence-based clinical practice guidelines (CPGs). However, until now, studies comparing the effect of digital education on CPGs have shown only a moderate difference in favor of digital education intervention [5], highlighting the need for novel and more powerful approaches. Actually, though the AIM literature proposes a quite large number of domain-independent CIG systems (see, e.g., the surveys [6–8]), such systems have been designed to provide *decision support* to physicians.

Few approaches in CIG literature specifically target *educational* tasks. The first proposal, mentioned in [55], suggests using a multipurpose CIG execution engine for education, though its architecture is too general to address specific educational tasks. More recently, AIM tools have been developed for training physicians on clinical guidelines. For instance, Real et al. [56] used decision tables based on guidelines for arterial hypertension to train residents, comparing their decisions with those of the tables. Riaño et al. [57] describe Shock-Instructor, a web-based tool that helps hospital residents learn about shock management through a knowledge base and case descriptions. Uribe-Ocampo et al. [58] introduced SIM-CIG, a video game for simulating antenatal healthcare scenarios and evaluating decision-making based on obstetric guidelines. Notably, the approaches in [56–58] are devoted to a specific domain. GLARE-Edu is the only CIG-based educational system which.

1. Is domain independent.
2. Provides verification/testing facilities.

Feature (1) grants that, with GLARE-Edu, education concerning different diseases can be homogeneously performed, adopting the same “conceptual map” representation and educational facilities. On the other hand, the educational impact of feature (2) has been already discussed in Sect. 2 above. Thus, GLARE-Edu constitutes a step forward in the panorama of CIG-based medical education.

The only evaluation of CIG-based educational system is reported in [57]. The study in [57] compares the two groups of learners (experimental and control) in terms of: (i) guideline adherence, (ii) accuracy in dose prescriptions, (iii) survival rate post-intervention, and (iv) frailty of the surviving cases. In light of current research, we can therefore compare [57] with our approach solely on aspect (i) (see Sect. 4.1), while leaving the other comparisons for future trials (see Sect. 4.2). Specifically, [57] reports a statistically significant improvement (p-value = 0.01) of 2% in guideline adherence.

Our preliminary experiment, however, indicates a 10% improvement in adherence related to activity-based actions, a 4% improvement in data requests, and a 9.5% improvement in staging-related decisions (i.e., clinical decisions), leading to an average improvement of at least 7–8%, without accounting for the imbalance in the number of actions across these types. Although the performance achieved with GLARE-Edu cannot be deemed statistically significant due to the limited number of learners involved, these findings nonetheless represent an interesting effect that will require further verification through the more extensive trial currently underway within the AI-LEAP project.

Notably, to the best of our knowledge, all the CIG-based approaches in the AIM literature are symbolic approaches, based on an explicit representation of medical knowledge. This solution seems quite natural, since CPGs are “golden standards” that must be elaborated, *specified and*,* above all*,* certified by dedicated teams of medical experts* (these requirements are, e.g., explicitly stated in the Italian law [59]). Given the availability (in textual CPGs) of such high-quality knowledge, the adoption of (explicit) knowledge representation and reasoning techniques looks natural and appropriate, since “*A central tenet of the symbolic paradigm is that intelligence results from the manipulation of abstract compositional representations*” [10]. Indeed, machine learning techniques can be used to discover from data the actual medical procedures adopted to manage specific diseases. In particular, a specific area of AI, termed ***process mining***, has (among the other) the specific task of ***discovering process models*** from data (more precisely, from the traces of the executions of processes) [60]. We are active in such an area of research [61, 62], but it is important to point out that the process models discovered by process mining considering the traces of activities performed on patients are ***not*** (officially recognized) clinical guidelines, since they may contain errors and deviations from the “golden standard”. Indeed, comparing the discovered process models with the CPG “golden standard” is an important activity, to identify deviations (which may be either due to errors in the execution of CPGs, or suggest new practices that experts can evaluate to possibly update the CPG golden standard) [60].

In general, we strongly believe that the symbolic and machine learning methodologies are highly complementary (consider, e.g., [10]), and can be fruitfully integrated in the CPG context. For instance, in our future work (see Sect. 6), we envision adopting machine learning techniques to refine and personalize case studies.

## Conclusions and Future Works

In this paper, we have described GLARE-Edu, a symbolic AI approach proposing an explicit and structured representation of CPGs and navigation, automated simulation and verification facilities to support medical education. GLARE-Edu educational impact is twofold, since it may support teachers both teachers’ activities (supervised use of the system) and learners’ autonomous study, providing them.

- A structured symbolic representation of CPGs at multiple levels of detail, and a navigation facility to explore it,
- The automatic adaptation of CPGs to case studies (i.e., automatic focusing on the part of the CPG needed for the case study at hand, through the automated simulation facility), and.
- A (self)verification facility.

The main original aspects of GLARE-Edu are the fact that it is (to the best of our knowledge) the first CIG-based educational system that.

- Is domain-independent.
- Provides verification and self-verification facilities.

Additionally, two major technical contributions of our approach are:

- The definition and description of a comprehensive architecture that integrates CPG acquisition, case study acquisition, and the navigation, automated simulation and verification facilities.
- The discussion about the extensions needed in order to move from a “traditional” CIG-based decision support system to an educational system (providing the above facilities).

In the short term, future work concerns mainly the large-scale experimental evaluation discussed in Sect. 4.2.

From the technical point of view, in our long-term research plan, we envision two main future developments. First of all, we plan to enhance our facility to acquire case studies with machine learning or generative AI techniques, in order to support the refinement and personalization of case studies on the basis of the traces provided by the application of the verification facility. Second, another significant future extension of GLARE-Edu will address exceptional patients, such as comorbid patients (i.e., patients suffering from two or more illnesses) [63]. Treating such patients presents a dual challenge for the physician, who must be capable of adhering to multiple guidelines simultaneously while detecting and managing potential interactions.

Building on our previous work concerning the management of comorbid patients [64, 65], our objective is to extend GLARE-Edu along several dimensions:

- First, GLARE-Edu must be equipped to handle case studies representing comorbid patients, including all the actions that modify CIGs to manage interactions.
- The automated simulation tool must be extended to show learners which parts of the different CIGs interact, how to classify interactions (e.g., interactions that enhance treatment, ones that cause toxicity), and how to apply interaction management strategies.
- The verification tool must be extended to enable learners to focus on different sections of the two CIGs and must ask them to identify and classify present interactions. The module will then ask learners to manage these interactions by correctly applying possible management approaches. Such extension is challenging, requiring reasoning concerning various knowledge sources (e.g., pharmacological, basic medical knowledge, ontological knowledge).

## Data Availability

The data that support the findings of this study are available from the corresponding author upon reasonable request.

## Footnotes

1 In artificial intelligence, **symbolic artificial intelligence** (also known as **classical artificial intelligence** or **logic-based artificial intelligence**) is the term for the collection of all methods in artificial intelligence research that are based on high-level symbolic (human-readable) representations of problems, logic and search [10].

2 The demo has been presented at ECAI’24, Doctoral Consortium.

3 Concerning CME courses, the accreditation process involves a set of steps: The course design, based on educational needs and clear objectives; The content evaluation, reviewed by a scientific training committee, which will work according to the Regional Council of December 20, 2019, No. 21–778; The accreditation application and evaluation, submitted to the accrediting body (i.e. Piedmont Region). In the case of positive final evaluation, the course is accredited and published on regional website [19]. Moreover, the quality of the CME courses is monitored in-itinere through event evaluations and the UNI EN ISO 9001:2015 quality system, which guarantees an appropriate organizational management system based on ISO policies and related procedures. Concerning ADE courses, course proposals must be evaluated by the President and Vice-president of the Degree program based on the contents quality, the objectives, the prerequisites required for an effective participation. ADE have to be scheduled to avoid any overlap with curricular activities. Finally, the course’s progress will be closely monitored to ensure it aligns with the original proposal in terms of quality and relevance.
